# Pediatric advocacy: Advancement in academic institutions

**DOI:** 10.1038/s41390-023-02997-1

**Published:** 2024-01-09

**Authors:** Sara M. Bode, Eimaan Anwar, Debra L. Best, Mona Patel, Lee S. Beers, Jeffrey M. Kaczorowski, Barry S. Solomon, Lisa J. Chamberlain

**Affiliations:** 1 Department of Pediatrics, Nationwide Children’s Hospital, Columbus, OH USA; 2 Department of Pediatrics, Stanford University School of Medicine, Palo Alto, CA USA; 3 Department of Pediatrics, Duke University School of Medicine, Durham, NC USA; 4 Department of Pediatrics, Children’s Hospital Los Angeles, Keck School of Medicine of USC, Los Angeles, CA USA; 5 Department of General and Community Pediatrics, Children’s National Health System, Washington, DC USA; 6 Department of Pediatrics, Golisano Children’s Hospital, Rochester, NY USA; 7 Department of Pediatrics, Johns Hopkins University School of Medicine, Baltimore, MA USA

## Abstract

- Children are facing many threats to their health today that require system change at a sweeping level to have real-world impact.
- Pediatricians are positioned as natural leaders to advocate for these critical community and policy changes.
- Academic medical center (AMC) leaders recognize the importance of this advocacy and clear steps can be taken to improve the structure to support pediatricians in their advocacy careers through faculty development and promotion, including standardized scholarly measurement of the outcomes.

Use of adaptive leadership strategies at all institutional levels can provide a framework to sustain advocacy activities around these challenging and complex health issues.

Academic pediatricians have a powerful voice for change as a unique constituency for children. They are also largely responsible for training the next generation of pediatric providers to become advocates for their patients and communities. The key questions we hope to answer in this paper are: (1) How can institutions support and encourage advocacy by academic pediatricians? And conversely, (2) How can academic pediatricians help facilitate system change toward advocacy scholarship?

## Need for advocacy

Children, adolescents, and families face an array of health-related challenges, many shaped by state or federal policies that may create or perpetuate racial inequities. The promising 15-year decline in childhood poverty to 11% in 2019 changed course during the COVID-19 pandemic. In January 2022, the overall child poverty rate rose to 17% with higher rates seen in children who identify as Black (25.4%) and Latino (23.9%).^1^ Similarly, it is projected that approximately 5 million children will lose health insurance with the “Medicaid unwinding” at the end of the public health emergency, with a disproportionate impact on Hispanic and Black individuals.^2,3^ Escalating rates of anxiety and depression coupled with limited access to mental health treatment and scarce inpatient beds led to the 2021 Declaration of National Emergency for Child and Adolescent Mental Health issued by the American Academy of Pediatrics, the American Academy of Child and Adolescent Psychiatry and the Children’s Hospital Association. Firearm-related deaths emerged as the leading cause of mortality in youth while threats to reproductive health care and gender-affirming care have become a harsh reality. These complex and varied threats to child well-being highlight the need for pediatricians to develop community and policy initiatives to elevate research-based best practices and mitigate the impact on children. The need for pediatricians to serve as child advocates has never been more pressing.

## Value of advocacy in academic pediatric departments

Unlike physicians who care exclusively for adults, pediatricians are well-positioned to advocate on behalf of children. Pediatricians see and bear witness to the impact of the social determinants of health on children. Pediatricians also enjoy uniquely high levels of trust, particularly because of the longitudinal continuity of care provided.^4^ Finally, pediatricians are able to contextualize the evidence-based recommendations when interacting with policymakers.

If we want pediatricians of the future to practice advocacy they must encounter it during their training, both in focused educational settings and being role-modeled in AMCs where most training occurs. Given the urgent need to advance the advocacy efforts on behalf of children, how do academic pediatricians align this critical work with AMCs? In an effort to understand the value placed on advocacy, we surveyed pediatric department chairs across the United States.^5^ Results indicate academic pediatric chairs felt increasing importance of advocacy in their departments over time, more than 75% felt it was important for faculty overall, 55% thought it was “important” or “very important” for promotion and 62% believed supporting advocacy was “important” or “very important.” Chairs expressed an abundance of barriers to advocacy activities by faculty in their departments, including sustainability for advocacy such as funding and protected time, and uncertainty around their role in advocacy as a pediatric department within larger health systems.^5^ Further work is underway to understand how health systems support and dissuade physician advocacy.

## Department role to increase value of advocacy

Pediatric departments can advance advocacy activities through alignment, training, and creating innovative funding opportunities and leadership positions. First, strategic planning should focus on aligning faculty advocacy endeavors with hospital and department missions. The use of shared language in the hospital community benefit report as well as annual Chair reports, allows for alignment between faculty efforts within larger missions, making a clearer case for investment in the work. Second, formal assistance is necessary as many faculty lack curricular training, mentorship, or protected time to engage in advocacy work. AMCs should provide faculty training and resources through professional development curricula, mentorship programs, and internal grant-funded opportunities. Investing in structural support for faculty advocacy activities will lead to sustainable advocacy. Third, once the framework and opportunities are established, advocacy efforts must also be aligned with not only traditional academic values but also traditional academic structures. This includes the development of leadership positions such as Vice Chairs and department advocacy leaders, which when complemented with a scholarly approach to the work, is inclusive of a pathway forward to promotion. Finally, departments can support the translation of advocacy work into faculty academic promotion using an advocacy portfolio (AP).^6,7^ Modeled after the success of the educator’s portfolio, the AP can provide a clear roadmap by incorporating advocacy work into the promotion domains of scholarship/research, education, and service/clinical.

One example involves Duke University School of Medicine Appointments, Promotion and Tenure that, in 2020, debuted a new framework for promotion at the faculty, department, and institutional level that values the traditional areas of primary focus (research, clinical, education) while also valuing the many “expressions of scholarship” of faculty that uphold institutional values.^8^ They define advocacy scholarship as “scholarly activity that promotes the social, economic, educational, and political changes that ameliorate threats to human health and advance the well-being of people.” As with traditional scholarship, work cited is required to highlight proof of excellence and evidence of a scholarly approach. Faculty identify advocacy-specific scholarly areas cited in the AP: advocacy engagement, community outreach, knowledge dissemination, advocacy teaching/mentoring, and advocacy leadership/administration. Additional forms of high-impact advocacy work beyond peer-reviewed manuscripts include co-authorship of policy statements/legislative briefs/consensus statements, legislative testimony, development of public health initiatives that become standard of care, participation in local and regional task forces, and establishment of community partnerships.

## Integrating advocacy across pediatrics

There is a need to develop a scholarly framework and advocacy metrics to standardize how this work is viewed across academic pediatrics to allow the field to evolve in an evidence-based way.^9^ Documenting quantifiable evidence of advocacy work enables appointment and promotion committee members to evaluate the scholarly quality of such work for advancement.^10^ Nerlinger et al describe the use of a logic model of demonstrating quality and quantity inputs to measure impacts and outcomes as one way to measure the scholarship of advocacy initiatives.^10^ A case example of using a logic model for advocacy in improving outcomes for asthma follows. Imagine a 5-year-old female with poorly controlled moderate persistent asthma, who lives in a household with mold and has inadequate access to her medications. This leads to increased utilization of emergency room and urgent care visits for asthma exacerbation with resultant hospitalization for status asthmaticus. The logic model maps inputs and outcomes, including the number of referrals made to community-based organizations, including medical-legal partnerships, to aid with remediating housing conditions for this child. Providing testimony for mold to be added to the list of state housing code violations is an example of high-impact advocacy. Working with managed care health plans to ensure access to evidence-based asthma medications demonstrates a second policy intervention. Additionally, systematically partnering with the community health workforce in asthma remediation is an example of community-engaged advocacy that can measurably improve patient outcomes. Finally, traditional dissemination of these advocacy efforts in abstracts, oral presentations, or publications is also possible. Using this stepwise approach is important because attribution of success in advocacy may take many years and an often-circuitous path, especially for legislative and policy efforts.

## Promoting long-term sustainability for health advocacy within your institution

Ultimately, faculty advocacy activities and scholarship require institutional support for effectiveness and sustainability. As advocacy is an emerging component of the academic health system model, long-term institutional buy-in will require both champions in senior leadership and grassroots efforts (Table 1). Medical education is increasingly embracing the integral role of health advocacy and the closely related importance of community collaboration as essential components in improving health systems and patient outcomes. In 2021, leaders from the Association of American Medical Colleges (AAMC) wrote “Embracing community collaborations as academic medicine’s fourth mission provides an opportunity to reimagine what optimal health can be—together….and make meaningful progress toward achieving health justice for all. Now is our time to act.”^11^ In order to promote changes that allow faculty to thrive as health advocates, institutions need to implement substantive changes and investments and/or expand their perspective on promotion, career development, and community engagement.

**Table 1:** Steps to Promote Advocacy in Your Institution.

RECOMMENDATIONS FOR ACADEMIC INSTITUTIONAL LEADERS
**1. Utilize strategic planning to align your hospital and department missions to value advocacy as a core component** Perform strategic planning in committee, including key stakeholders in academic pediatrics, to present a shared vision of advocacy and orient faculty in their advocacy work
**2. Provide structural support for faculty to encourage advocacy** ◼ Opportunities for formal training and professional development to enhance the advocacy skills of faculty and encourage involvement ◼ Infrastructure for mentorship amongst faculty to endorse learning and increase unity ◼ Protected time for faculty to ensure advocacy is an integral role in their careers ◼ Provide formal funding for advocacy activities
**3. Use an advocacy portfolio** ◼ A new framework for promotion that values advocacy scholarship to recognize and reward work to address social determinants of health for children and families

**RECOMMENDATIONS FOR PEDIATRICIANS**
**1. Understand your health system infrastructure** ◼ What role does advocacy currently play in your department? ◼ What resources are available to you to support your advocacy efforts? ◼ Meet with your department chair to understand how this aligns with your academic track
**2. Promote an advocacy portfolio** ◼ Does your institution’s appointments and promotions committee use advocacy metrics? ◼ Who in your department can you work with to push for advocacy scholarship?
**3. Foster collaboration in your institution and beyond** ◼ Build a relationship with your hospital’s government relations team ◼ Create alliances with elected officials and partner advocates

Institutional leaders at all levels may face barriers to achieving this vision and need to rely on various theories and styles of change management. One particularly relevant approach, adaptive leadership, is designed to address complex and longstanding challenges and is centered on building capacity across all organizational levels.^12^ This approach leverages past successes while at the same time prioritizing innovation and diversity of thought, staff, and expertise; it recognizes that any successful “adaptation” preserves what is essential, reorganizes what no longer best meets an organization’s needs, and creates new opportunities to allow an organization or individual to thrive.^12^

Using this framework, several examples follow of activities that may be particularly impactful in advancing a sustainable commitment to advocacy within academic institutions, though there are many other potentially effective activities and strategies.

- Preserving what is essential: First and foremost, it is important to identify and articulate the overarching sense of purpose and how day-to-day activities are influenced by these guiding principles. Ensuring that their team is reminded of and centered on this sense of purpose will prepare advocates for the potentially hard work ahead and ensure they stay true to your mission. It is critical for advocates to understand and convey that successful advocacy results in systemic change to benefit children and families, their community, and potentially their institution or practice, including new or expanded funding.
- Better meeting an organization’s needs with existing resources: By focusing on three key areas—increased relationship building with elected officials and partner advocates, enhancing communication (including with media), and aligning existing resources – leaders can strengthen existing infrastructure and provide more focus on desired activities and outcomes. To achieve success in these areas, it is useful to think about who potential champions are and/or who is likely to be influential in achieving their goals work to better understand their priorities and how they may align with the advocate’s vision for change. Sometimes relatively minor adjustments within an organization can have a large impact.
- Creating new opportunities: The cultivation of supplemental funding sources can provide opportunities for innovation and be an important lever for achieving buy-in and leveraging additional institutional support. Some funding opportunities may be solely focused on health advocacy and/or community engagement, but it is also useful to look for opportunities to partner with others and enhance their work.

Successful advocacy is demanding and complex, as are the multitude of problems we are challenged to address, from gun violence to mental health. Pediatrics must always maintain a priority focus on what is best for children and families and be ready to adopt innovative approaches to advance child well-being. Leveraging institutional leadership support, providing a framework and pathway for faculty advancement through a scholarly approach, and strengthening partnerships, funding, and communications will continue to advance successful advocacy efforts.
